# More blastocysts are produced from fewer oocytes in ICSI  compared to IVF – results from a sibling oocytes study and definition of a new key performance indicator

**DOI:** 10.1186/s12958-021-00804-2

**Published:** 2021-07-26

**Authors:** Sandrine Chamayou, Carmen Ragolia, Carmelita Alecci, Giorgia Storaci, Simona Romano, Roberta Sapienza, Elena Maglia, Annalisa Liprino, Clementina Cardea, Michele Fichera, Antonino Guglielmino

**Affiliations:** 1 Unità di Medicina della Riproduzione - Centro HERA, via barriera del bosco, n. 51/53, Sant’Agata Li Battiati, Catania Italy; 2 Unit of Gynecology and Obstetrics-Department of General Surgery and Medical Surgical Specialties, University of Catania, Catania, Italy

**Keywords:** Blastocyst, ICSI, IVF, Key performance indicator, Sibling oocyte, Split insemination

## Abstract

**Background:**

Which fertilization method, between ICSI and IVF in split insemination treatments, has the highest clinical efficiency in producing clinically usable blastocyst?

**Methods:**

211 infertile couples underwent split insemination treatments for a non-severe male factor. 1300 metaphase II (MII) oocytes were inseminated by conventional IVF and 1302 MII oocytes were micro-injected with the same partner’s semen. Embryo development until blastocyst stage on day V and clinical outcomes were valuated trough conventional key performance indicators (KPI), and new KPIs such as blastocyst rate per used MII oocytes and the number of MII oocytes to produce one clinically usable blastocyst from ICSI and IVF procedures.

**Results:**

The results were  globally analyzed and according to ovarian stimulation protocol, infertility indication, and female age. The conventional KPI were online with the expected values from consensus references. From global results, 2.3 MII oocyte was needed to produce one clinically usable blastocyst after ICSI compared to 2.9 MII oocytes in IVF. On the same way, more blastocysts for clinical use were produced from fewer MII oocytes in ICSI compared to IVF in all sub-groups.

**Conclusions:**

In split insemination treatments, the yield of clinically usable blastocysts was always superior in ICSI compared to IVF. The new KPI "number of needed oocytes to produce one clinically usable embryo" tests the clinical efficiency of the IVF laboratory.

## Key Message

In split insemination study treatments, the yield of clinically usable blastocysts is superior in ICSI compared to IVF. The new KPI “number of needed oocytes to produce one clinically usable embryo” tests the clinical efficiency of the IVF laboratory.

## Introduction

The two main in vitro fertilization techniques are conventional In Vitro Fertilization (IVF) [1] and Intra-Cytoplasmic Sperm Injection (ICSI) [2, 3]. Conventional IVF solves gynaecological infertility such as tubal factor, endometriosis, anovulation, unexplained infertility, and moderate male factor [4]. ICSI solves severe male factor [2], unexplained infertility with previous fertilization failure [5], is commonly applied in case of reduced oocyte yield [6] and when thawed oocytes are used [7]. Consequently, and worldwide, we observe an increasing use of ICSI over IVF that reached 71.3% of fresh in vitro treatments [8]. In Italy, conventional IVF with fresh and non-donated gametes was applied in only 15.2% of the assisted fertilization treatments in 2019 [9].

Since the early 2000’ and in different IVF units, the split insemination strategy has been applied to compare IVF and ICSI results from oocytes produced from the same ovarian stimulation (sibling oocytes) [10, 11]. Through this strategy, it is possible to verify if gametes are competent to fertilize by themselves in IVF or if they need to be assisted by ICSI. In the meantime, the embryo transfer should be ensured from embryos produced in ICSI.

Different groups have compared IVF and ICSI results and efficacy on sibling oocytes in different groups of patients. The Key Performance Indicators (KPIs) applied to compare the two fertilization methods were fertilization and cleavage rates, embryo quality, the percentage of transferred and frozen embryos on zygote number, and clinical outcome measurements such as implantation and clinical pregnancy rates. Different studies found the two fertilization methods comparable and suggest applying IVF when possible [12–14] even when the fertilization rate was higher in ICSI [11, 15–17]. In all cases, the authors tested the goodness of IVF and ICSI procedures in their lab from a technical perspective. The expected values of the laboratory KPIs are currently reported in consensus references [18–20]. On the other side, the real efficiency of each fertilization procedure in producing an embryo for clinical use and a live baby born for the studied populations was not estimated in the above studies. Consequently, and based on systematic reviews and meta-analyses of previously published data, the (non-)advantage of ICSI over IVF for non-severe male factor can still be considered an open question [21, 22].

To evaluate the real reproductive prospective of ICSI and IVF, we need to compare the two fertilization methods from a clinical point of view using laboratory data in split insemination treatments. New KPIs such as the "number of metaphase II (MII) oocytes needed to produce one clinically usable blastocyst" will help in understanding which of the two methods produces the highest number of clinically usable blastocysts for fresh and postponed clinical use.

In our center and since 1997, split insemination treatments are applied in accordance with the physician and the patients based on their previous infertility history and the male factor status. Since 2011, embryo culture is performed under time-lapse monitoring in our IVF lab [23]. Consequently, data from split insemination treatments are easily accessible and can be retrospectively analyzed.

In the present study, we compared retrospective in vitro results and clinical outcomes after blastocyst transfer on day V of split insemination treatments (conventional IVF and ICSI). The results were analyzed in their globality and according to ovarian stimulation protocol, infertility indication, and female patient age group. To perform these analyses, conventional and new KPIs were used to test each fertilization method's efficiency in producing the highest number of blastocysts for clinical use.

## Materials and methods

All procedures were approved by our Institutional Review Board. All participants gave written consent on all aspects of the treatment after having been informed.

### Patients and ovarian stimulation

Between March 2011 and March 2020, 334 couples underwent split insemination treatments. For the present study, only couples in which embryo-culture was continued until day V were selected. The fate of embryo to transfer or freezing was based on embryo morphology and morphokinetic only, and not on fertilization method. Furthermore, we included only cases in which the final fate of each blastocyst produced from ICSI or IVF was determined: embryo-transfers with only ICSI embryos; embryo-transfers with only IVF embryos; and embryo-transfers with embryos from both ICSI and IVF and with the same fate (none implanted or both implanted). The embryo-transfers with embryos from both ICSI and IVF in which only one embryo implanted were excluded from the present study. Consequently, 211 cases were selected.

The infertility indications were anovulation (24 couples), endometriosis (14 couples), male factor (20 couples), polycystic ovarian syndrome (5 couples), tubal factor (57 couples), and unexplained infertility (91 couples). Female patients were aged between 19.8 and 44.3 years (mean age: 33.9), and basal FSH on Day III was between 2.7 and 12.8 IU/l (mean 4.5 IU/l—Immulite 2000, Siemens-Germany). Karyotype was normal for all patients. To perform split insemination treatment, the semen parameters needed to be suitable to perform conventional IVF. Patients with severe oligotheratoastenozoospermia were excluded.

Two different protocols of ovarian stimulations were carried out. In the long protocol, agonist (gonadotrophin-releasing hormone-GnRHa, Suprefact: Hoechst Marion Roussel Deutschland GmbH, Frankfurt, Germany) started in the luteal phase and was followed by delay administration of recombinant FSH (Gonal-F: Merck-Serono, London, UK or Puregon, MSD, Franklin Lakes, USA) and LH (Luveris: Merck-Serono) after down-regulation. In the short protocol with an antagonist, ovarian stimulation was performed by administering recombinant FSH and LH from cycle Day III. When the leading follicle reached 14 mm in diameter, the antagonist was daily added with a dosage of 0.25 mg/day (Cetrotide: Merck-Serono, London, UK) until triggering. In both protocols, initial doses were 150–300 IU/day for FSH and 75–150 IU/day for LH. Triggering was done with HCG 10,000 IU (Gonasi: AMSA, Italy). Vaginal ultrasound-guided aspiration of oocyte − cumulus complexes was performed 35 h after human chorionic gonadotrophin administration.

### Split insemination (ICSI and IVF) and embryo culture

After oocyte retrieval, the cumulus-oocyte complexes (COC) were randomly divided into two groups. Gamete preparations were previously described [2, 3, 24]. In the first group and after cumulus cells elimination, MII oocytes were micro-injected with the partner's freshly ejaculated spermatozoa three hours after oocyte retrieval, as previously described [24]. In the meantime, and in IVF, each COC was inseminated with 25.000 motile spermatozoa from the same partner’s sperm sample for 3 h. After 3 h, cumulus cells were gently removed, and the meiotic state of inseminated oocytes were evaluated with the observation of the first polar body. All the key performance indicators based on the number of MII oocytes in IVF were calculated on the number of MII oocytes at that time.

After micro-injection in ICSI and incubation with partner’s sperm in IVF, the cell in vitro culture was placed in 25 ul of continuous, single culture complete medium with human serum albumin (Irvine Scientific, Santa Ana, USA) under mineral oil and in automated incubators with 5% CO2, 5% O2 at 37 °C, fitted with time-lapse imaging acquisition (Embryoscope, Unisense, Aarhus—Denmark). The entire embryo development has been followed and analyzed. During incubation in the Embryoscope, seven plane focal images were generated each 20 min and recorded.

In all the present cases, the number of available embryos at cleavage stage (day II-III) was superior to the number of embryos decided to be transferred, and the morphokinetic parameters matched with the predictive values to reach blastocyst stage previously established in our lab conditions [23]. Consequently, and in accordance with the patients' and physicians' decisions, embryo culture was continued until blastocyst stage for embryo transfer with/without surplus blastocyst vitrification.

After embryo transfer on day V, clinical pregnancy was ascertained by observation of fetal heartbeat in the successive weeks. Pregnancies were followed until birth.

### Blastocyst vitrification

The surplus blastocysts were vitrified according to the vitrification protocol previously described [24, 25].

### Key performance indicators

The conventional KPIs in assisted reproductive techniques were applied: fertilization rate, cleavage and blastocyst rates calculated per zygote, and implantation rate calculated per transferred embryos [18, 26].

Live birth rate was calculated per transferred embryos.

The new KPIs were the blastocyst rate per (microinjected or inseminated) MII oocyte, and the number of MII oocytes needed to produce one clinically usable blastocyst on day V (to transfer or to freeze).

All rates were compared between IVF and ICSI on sibling gametes.

### Statistical analysis

As the size of studied samples was superior to 25, the statistical significance of rates was tested by z test at different levels of significance (*p* < 0.05, 0.01, 0.001). The normal distribution of data was assumed. The null hypothesis was no difference between the checked rates.

## Results

In vitro results and clinical outcomes were analyzed in their totality and according to ovarian stimulation protocol, infertility indication, and female age.

## Global results

### In vitro results

A total of 2602 COC were retrieved (mean of 12.3 COC per OPU) from which 1302 COC were used for ICSI and 1300 for IVF. After decumulation, it resulted that 900 MII oocytes were inseminated by conventional IVF (mean number: 4.3) and 904 MII oocytes were micro-injected by ICSI (mean number: 4.3). The fertilization rate was statistically superior in ICSI (729/904, 80.6%) compared to IVF (596/900, 66.2%; *p* < 0.001). There was no statistical difference in the cleavage rate (709/729, 97.3% in ICSI and 573/596, 96.1% in IVF; *p* > 0.05) and blastocyst rate per zygote on day V (399/729, 54.7% in ICSI and 309/596, 51.8% in IVF; *p* > 0.05) between the two fertilization techniques.

According to the new KPIs, the blastocyst rate calculated on MII oocyte was statistically superior in ICSI (399/904, 44.1%) compared to IVF (309/900, 34.3%, *p* > 0.001). Consequently, the number of oocytes needed to produce one clinically usable blastocyst was a mean of 2.3 after ICSI and 2.9 after IVF.

### Clinical outcomes

Of the 188 ICSI-blastocysts transferred on Day V, 80 implanted (42.6%) leading to 72 live births (38.3%). Of the 178 IVF-blastocysts transferred on day V, 69 implanted (35.4%, *p* > 0.05) leading to 63 live births (35.4%; *p* > 0.05) (Table 1).

**Table 1:** Comparison of in vitro results after blastocyst transfers after ICSI and IVF on sibling oocytes in split insemination cycles

	**ICSI**	**IVF**	***p***	**TOTAL**
n. cycles	211			**211**
n. COCs	1302	1300		2602
n. MII micro-injected or inseminated oocytes	904	900		1804
n. zygotes	729	596		1325
**fertilization rate**	**80.6**	**66.2**	** < 0.001**	**73.4**
n. cleaved embryos	709	573		1282
**cleavage rate**	**97.3**	**96.1**	**NS**	**96.8**
n. blastocysts	399	309		708
**blastocyst rate/ zygote**	**54.7**	**51.8**	**NS**	**53.4**
**blastocyst rate/ MII oocyte**	**44.1**	**34.3**	** < 0.001**	**39.2**
**n. MII oocytes per usable blastocyst**	**2.3**	**2.9**	** < 0.001**	**2.5**
n. frozen blastocysts	211	131		342
n. transferred blastocysts	188	178		366
n. implanted blastocysts	80	69		149
**implantation rate**	**42.6**	**38.8**	**NS**	**40.7**
n. live births	72	63		135
**live birth rate**	**38.3**	**35.4**	**NS**	**36.9**

### According to ovarian stimulation protocol, infertility indication and female patient age

The in vitro results and clinical outcomes after ICSI and IVF on sibling oocytes were divided in sub-groups and analyzed according to ovarian stimulation protocol in 122 couples (mean female age: 34.3) after ovarian stimulation with long protocol and 89 couples (mean female age: 33.6) after short protocol; according to infertility indication in 91 couples with unexplained infertility (mean female age: 34.9), 20 couples with male infertility (mean female age: 31.6) and 100 couples with female infertility (anovulation, endometriosis, polycystic ovarian syndrome, tubal factor) (mean female age: 33.5) and according to female patient age (159 female patients aged between 19 and 37 years old, and 52 aged between 38 and 45 years old) (see Table 2).

**Table 2:** Comparison of in vitro results after ICSI and IVF on sibling oocytes in split insemination cycles and according to ovarian stimulation protocol, infertility indication and female age

	**TOTAL**	**Ovarian stimulation protocol**						**Infertility**									**Female age**					
		**Long**			**Short**			**Unexplained**			**Male**			**Female**			**19–37 years old**			**38–45 years old**		
		**ICSI**	**IVF**	***p***	**ICSI**	**IVF**	***p***	**ICSI**	**IVF**	***p***	**ICSI**	**IVF**	***p***	**ICSI**	**IVF**	***p***	**ICSI**	**IVF**	***p***	**ICSI**	**IVF**	***p***
n. cycles	**211**	122			89			91			20			100			159			52		
n. COCs	2602	571	570		731	730		540	539		128	128		634	633		1034	1033		268	267	
n. micro-injected or inseminated MII oocytes	1804	396	395		508	505		374	374		89	89		441	437		717	716		187	184	
n. zygotes	1325	330	279		399	317		309	248		69	55		351	293		584	470		145	126	
**fertilization rate**	**73.4**	**83.3**	**70.6**	** < 0.001**	**78.5**	**62.8**	** < 0.001**	**82.6**	**66.3**	** < 0.001**	**77.5**	**61.8**	** < 0.05**	**79.6**	**67.0**	** < 0.001**	**81.5**	**65.6**	** < 0.001**	**77.5**	**68.5**	** < 0.05**
n. cleaved embryos	1282	326	265		383	308		299	239		65	54		345	280		567	448		142	125	
**cleavage rate**	**96.8**	**98.8**	**95.0**	** < 0.01**	**96.0**	**97.2**	**NS**	**96.8**	**96.4**	**NS**	**94.2**	**98.2**	**NS**	**98.3**	**95.6**	** < 0.05**	**97.1**	**95.3**	**NS**	**97.9**	**99.2**	**NS**
n. blastocysts	708	222	158		177	151		177	129		39	30		183	150		327	243		72	66	
**blastocyst rate/ zygote**	**53.4**	**67.3**	**56.6**	** < 0.01**	**44.4**	**47.6**	**NS**	**57.3**	**60.9**	**NS**	**56.5**	**54.5**	**NS**	**52.1**	**51.2**	**NS**	**56.0**	**51.7**	**NS**	**49.7**	**52.4**	**NS**
**blastocyst rate/ MII oocyte**	**39.2**	**56.1**	**40.0**	** < 0.001**	**34.8**	**29.9**	** < 0.05**	**47.3**	**24.5**	** < 0.001**	**43.8**	**33.7**	**NS**	**41.5**	**34.3**	** < 0.05**	**45.6**	**33.9**	** < 0.001**	**38.5**	**35.9**	**NS**
**n. oocytes per usable blastocyst**	**2.5**	**1.8**	**2.5**	** < 0.001**	**2.9**	**3.3**	** < 0.05**	**2.1**	**2.9**	** < 0.001**	**2.3**	**3.0**	** < 0.05**	**2.4**	**2.9**	** < 0.05**	**2.2**	**2.9**	** < 0.001**	**2.6**	**2.8**	**NS**
n. frozen blastocysts	342	103	47		108	84		96	52		21	13		94	66		190	115		21	16	
n. transferred blastocysts	366	119	111		69	67		81	77		18	17		89	84		137	128		51	50	
n. implanted blastocysts	149	49	40		31	29		32	30		8	7		40	32		69	58		11	11	
**implantation rate**	**40.7**	**41.2**	**36.0**	**NS**	**44.9**	**43.3**	**NS**	**39.5**	**39.0**	**NS**	**44.4**	**41.2**	**NS**	**44.9**	**38.1**	**NS**	**50.4**	**45.3**	**NS**	**21.6**	**22.0**	**NS**
n. live births	135	43	37		29	26		31	29		7	5		34	29		63	55		9	8	
**live birth rate**	**36.9**	**36.1**	**33.3**	**NS**	**42.0**	**38.8**	**NS**	**38.3**	**37.7**	**NS**	**38.9**	**29.4**	**NS**	**38.2**	**34.5**	**NS**	**46.0**	**43.0**	**NS**	**17.6**	**16.0**	**NS**

### In vitro results

On a global view, the comparability of ICSI and IVF in vitro results were similar through the analyses of ovarian stimulation protocol, infertility indication and female patient age group.

Fertilization rate was always significantly superior in ICSI compared to IVF in all sub-groups. Cleavage rate was equivalent after ICSI and IVF, except for long protocol and female indication sub-groups where it resulted significantly superior in ICSI (*p* < 0.01). Blastocyst rates calculated on zygote were equivalent in both techniques, except for long protocol sub-group where it was significantly superior in ICSI (*p* < 0.01).

According to the new KPIs, blastocyst rate per MII oocyte was superior in ICSI for all groups, reaching statistical significance in long and short protocols, unexplained infertility, female infertility and 19–37 years old groups. Consequently, the micro-injected or inseminated MII oocytes needed to obtain a viable blastocyst on day V, was always inferior in ICSI compared to IVF. It varied from 1.8 in ICSI after long ovarian protocol to 3.0 in IVF for moderate male factor.

All data are resumed in Table 3.

**Table 3:** Number of MII oocyte per fertilization method to produce one clinically usable embryo

n. MII oocytes per usable embryo	Transfer at blastocyst stage			
	**N**	**ICSI**	**IVF**	***p***
**TOTAL**	211	2.3	2.9	˂0.001
**OVARIAN STIMULATION PROTOCOL**				
** Long protocol**	122	1.8	2.5	˂0.001
** Short protocol**	89	2.9	3.3	˂0.05
**INFERTILITY INDICATION**				
** Unexplained infertility**	91	2.1	2.9	˂0.001
** Male infertility**	20	2.3	3.0	˂0.05
** Female infertility**	100	2.4	2.9	˂0.05
**FEMALE PATIENT AGE**				
** 19–37 years old**	159	2.2	2.9	˂0.001
** 38–45 years old**	52	2.6	2.8	NS

### Clinical outcomes

As for in vitro results, clinical outcomes followed the comparability of ICSI, and IVF in vitro results were similar through the analyses of ovarian stimulation protocol, infertility and indication and female age. In all sub-groups, the implantation rate and live birth rate calculated per transferred embryo were comparable.

## Discussion

In the present study, we compared in vitro results and clinical outcomes in treatments in which half of the oocytes from the same ovarian stimulation were inseminated in conventional IVF, and half of the oocytes were micro-injected with the same partner’s sperm in ICSI. The effects of fertilization methods on fertilization rate, embryo development, and competence to lead a live birth were studied.

The results were analyzed on their globality and successively according to the ovarian stimulation protocol, infertility indication, and female age. In the female age sub-group, the division was made between 37 and 38 years old because clinical outcomes and embryo implantation decrease drastically from 38 years old due to increasing embryo aneuploidy [27]. As a mandatory condition for the present study, the number of MII oocytes used for IVF and ICSI was equal and the fate of each transferred blastocyst was known.

To compare the results, conventional KPIs were applied such as fertilization rate, cleavage and blastocyst rates calculated on the number of zygotes, implantation rate and live birth rate [18, 26], and new KPIs were proposed. Analysis by conventional KPI from global data and data from sub-groups (ovarian stimulation protocol, infertility indication and female age), fertilization always resulted statistically superior in ICSI compared to IVF while cleavage rate, known implantation rate, and known live birth rates were comparable in ICSI and IVF. Blastocyst rate calculated on zygote was comparable in ICSI and IVF with statistical significance in the long ovarian stimulation protocol sub-group. All these KPIs are typically used to check IVF laboratory performance [18], even if implantation and live birth rates also depend on clinical procedures such as embryo transfer and uterine receptivity. The present conventional KPI values in ICSI and IVF were concordant with the expected values [18].

In concordance with our results analyzed in their globality and by sub-groups, previous authors applying split insemination found significant higher fertilization rate in ICSI compared to IVF [11, 15–17, 28], comparable cleavage rate [10–12, 14, 15, 20], no statistical difference in blastocyst rate [11, 13], comparable implantation [10, 12] and live birth rates [13, 16].

Nevertheless, after applying conventional KPIs to test our data, we did not feel that we were testing the true efficiency of each fertilization method from the global data and in each sub-group. Consequently, it was decided to test other KPIs (blastocyst rate calculated on MII oocyte, the number of oocytes needed to produce one embryo for clinical use) that could be more informative on the true potential on ICSI and IVF in obtaining a live birth. Which fertilization method of ICSI and IVF leads to the highest number of embryos for clinical use and for which group of patients? While the previous conventional KPI tested laboratory performance, the new KPIs tested the technique and in vitro procedures on a clinical and patient group perspective *inside* the lab. From our data and as a consequence of the highest fertilization rate in ICSI and the comparability of cleavage rate between ICSI and IVF, the blastocyst rate calculated on used oocytes resulted superior in ICSI compared to IVF. On the same way, the number of MII oocytes needed to produce one blastocyst for clinical use was inferior in ICSI. This difference was always statistically significant except for the 38–45 years old female patients’ group. This last result was in accordance with previous studies [19, 20] in which ICSI was shown *to not improve* reproductive outcomes for the couples with female patients over 38 years old [20] or over 39 [19]. From the present data, ICSI always has a higher efficiency compared to IVF due to a higher number of blastocysts available for direct transfer, freezing or other use (biopsy for preimplantation genetic testing). It was calculated that 26% (0.6/2.3) of surplus MII oocytes were needed to produce one blastocyst compared to ICSI. This value reached 38% in case of unexplained infertility and long ovarian stimulation protocol (respectively (0.8/2.1 and 0.7/1.8). Our results follow with Yovich et al*.* [17] that found a higher number of embryos to transfer in the ICSI group (2.5 versus 1.8 in IVF) in patients with mainly male factor issues. Mathematically, the highest number of clinically usable embryos in ICSI would increase the cumulative clinical rates.

The research of the best KPI to test ART efficiency and safety remains a subject of study. Different parameters are available to assess clinical management [29, 30] and laboratory performance and stability  [18, 31]. However, once the IVF laboratory has been monitored as a stable "tool" thanks to quality controls, it can be used to test procedures such as fertilization methods to produce a maximum of embryos for clinical use as done here. The comparison study of split insemination cycles from the same gamete cohorts (oocytes and spermatozoon) in the same ovarian cycle eliminates any clinical variants such as ovarian stimulation and responses that would be introduced in a non-sibling study  [32–35]. The definition of the new KPI “number of MII oocytes to produce one clinically usable embryo” is online with Fisher and Scott that underlined the need for simple metrics to define fertilization success rates [34].

Previous authors applying split insemination reported data from embryo development until cleavage stage [10, 12, 15, 16] or on a minor number of cases [11, 13, 14, 18, 28]. The present study is the first one focused mainly on blastocyst stage, and on such a high number of cases that could also be analyzed according to different clinical parameters.

Because fewer embryos are produced in IVF compared to ICSI even with no male indication, conventional IVF and split insemination cycles should be carefully proposed based on the number of oocytes and the probability to produce embryos to transfer or to freeze. According to the number of MII oocytes available, the physician can calculate the expected number of embryos based on the laboratory data.

## Conclusion

As per recent literature [36] and from the present study, clinical outcomes such as live-birth rates are comparable in ICSI and IVF in split insemination cycles. However, ICSI is more efficient in producing the highest number of blastocysts from the minimum number of MII oocytes compared to IVF in all patient groups and in particular for each ovarian stimulation protocol, infertility indication, or female age group. For the patients, the production of more embryos from one ovarian stimulation increases the chance of obtaining a pregnancy and a live birth. The long time effect on an increased cumulative live birth rate should confirm the present results. The novel KPI "number of MII oocytes to produce one clinically usable embryo" is an indicative parameter to test the clinical efficiency of ART procedures in the IVF lab.

## Data Availability

Not applicable.

## Availability of data and materials

All relevant data are within the text.
